# Complex roles of filamin-A mediated cytoskeleton network in cancer progression

**DOI:** 10.1186/2045-3701-3-7

**Published:** 2013-02-06

**Authors:** Jingyin Yue, Steven Huhn, Zhiyuan Shen

**Affiliations:** 1 Department of Radiation Oncology, The Cancer Institute of New Jersey, Robert Wood Johnson Medical School, New Brunswick, NJ, 08903, USA; 2 Current address: Molecular Pharmacology and Chemistry Program, Memorial Sloan-Kettering Cancer Center, New York, NY, 10065, USA

**Keywords:** Filamin-A, ABP-280, Actin filament, Cytoskeleton, DNA repair, Metastasis

## Abstract

Filamin-A (FLNA), also called actin-binding protein 280 (ABP-280), was originally identified as a non-muscle actin binding protein, which organizes filamentous actin into orthogonal networks and stress fibers. Filamin-A also anchors various transmembrane proteins to the actin cytoskeleton and provides a scaffold for a wide range of cytoplasmic and nuclear signaling proteins. Intriguingly, several studies have revealed that filamin-A associates with multiple non-cytoskeletal proteins of diverse function and is involved in several unrelated pathways. Mutations and aberrant expression of filamin-A have been reported in human genetic diseases and several types of cancer. In this review, we discuss the implications of filamin-A in cancer progression, including metastasis and DNA damage response.

## Introduction

The cytoskeleton, a complex network of protein fibers in eukaryotic cells, provides a dynamic structural framework that is crucial for maintaining normal cell activity, including cell shape, cellular motion, division, and intracellular transport among other processes [1-3]. Eukaryotic cells contain three main types of cytoskeletal filaments: microfilaments, intermediate filaments, and microtubules [1,3]. Microfilaments (also called actin filaments or F-actin) are composed of linear polymers of actin subunits that form the thinnest filaments of the cytoskeleton. The actin filament is a polar macromolecule characterized by the elongation of one filament end coupled with shrinkage at the other. This dynamic interplay generates force and causes net movement of the intervening strand [4]. Actin filaments also act as tracks for the movement of myosin molecules that attach to the microfilament and "walk" along them [5]. In addition, actin filaments cross-link into bundles to form the dynamic actin cytoskeletal network, which is in turn finely tuned by multiple families of cytoskeletal proteins [6], called actin binding proteins. These proteins typically share a conserved, α-actinin-like F-actin binding domain (ABD) [7-9].

**Figure 1:**
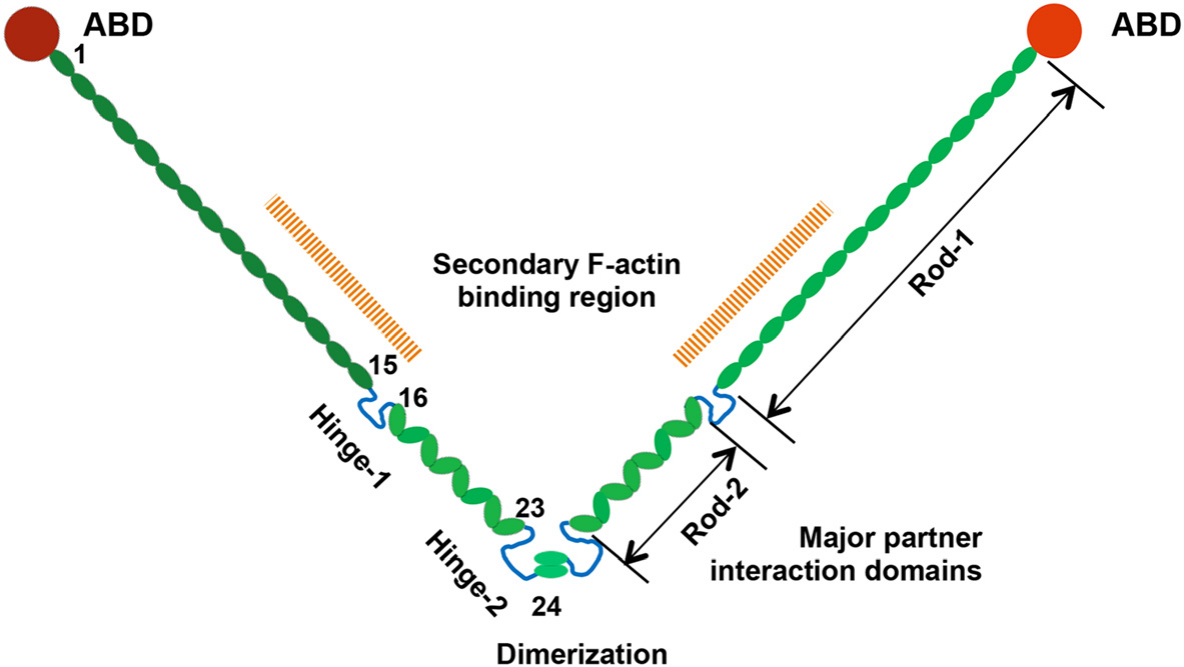
**Structure of human filamin-A protein.** Filamin-A is a V-shaped homodimer. Each monomer is a protein of 2647 amino acids that contains 24 tandem repeats. Each repeat contains ~96 amino acids. Filamin-A can be divided into 3 major domains: F-Actin-Binding Domain (ABD), the filamin-A Rod regions: Rod-1 containing repeat 1–15 and Rod-2 containing repeats 16–23; C-Terminal Domain (FCTD) of repeats 16–24, containing major partner interaction domains. In the FCTD, a defined C-terminal filamin-A dimerization domain of ~65 amino acids is located in repeat 24, and a filamin-A hinge regions of about 34 amino acids is located between repeat 23 and 24, and between repeat 15–16

One actin binding protein that contains the actinin-like F-actin binding domain is the filamin family. This family is composed of three homologous proteins (FLNA, FLNB, and FLNC) that are products of different genes and their mRNA splice variants [10]. The three filamin genes are highly conserved and filamin proteins exhibit 60-80% overall amino acid identity, with the greatest divergence observed at the two hinge regions, sharing 45% identity [11]. Filamin-A (FLNA), also known as human actin-binding protein 280 (ABP-280) or filamin-1, is encoded by the X-linked gene *FLNA*[12,13]. As shown in Figure 1, the human filamin-A is a homodimer with large subunits of 280KD, forming a V-shaped structure [11,14-16]. At the NH2 terminus of the monomer, there is an actin-binding domain (ABD), followed by 24 tandem repeats of ~96 amino acids in length. Between repeats 15 and 16, there is a hinge domain, and repeat 24 is separated from repeat 23 by a second hinge domain. The last 65 amino acids of repeat 24 mediates the dimerization of filamin-A subunits [14].

Filamin-A binds and cross-links cortical actin filaments into a dynamic three-dimensional structure through its N-terminal actin-binding domain [17,18]. In addition to filamentous actin, filamin-A interacts with more than 60 functionally diverse cellular proteins, including trans-membrane receptors, signaling molecules, DNA damage repair proteins. These diverse interactions suggest that filamin-A is a key component of a versatile signaling scaffold complex [15,17-19]. In this review, we discuss the role of filamin-A in human diseases, with an emphasis on cancer.

### Filamin-A mutations and human genetic diseases

Due to its versatile function in regulating cell motility and signaling, defects in the *FLNA* gene have been demonstrated as the cause for a wide range of developmental malformations involving the brain, bone, limbs, and heart [10,19]. Periventricular nodular heterotopia (PNH) is a well characterized neuron development disorder, mainly caused by loss-of-function mutations in *FLNA* gene [20-25], while other possible genetic contributions cannot be excluded [26-29]. In patients affected by PNH, clusters of grey matter along the ventricles consisting of neurons fail to migrate to the cortex during prenatal development. X-linked PNH is mainly confined to females, indicating that FLNA null mutations in males are predominantly associated with prenatal death. In addition, PNH patients can co-present abnormal cerebral migration with other brain, skeletal, or visceral abnormalities [30-35], while higher chance of *FLNA* mutation has been implicated as the causes for these cases. Furthermore, clustered missense mutations in *FLNA* have been identified in a diverse spectrum of congenital malformations in humans [19,36], including otopalatodigital syndrome (OPD), frontometaphyseal dysplasia (FMD) and Melnick–Needles syndrome (MNS). Moreover, using a familial and genealogical survey, it was discovered that missense mutations in *FLNA* were the likely genetic cause of familial cardiac valvular dystrophy [33,37,38].

The importance of filamin-A controlled cell adhesion and migration in human development has been confirmed using mouse models and established cell lines of filamin-A deficiency [39-43]. These approaches have been successful in reconstructing the phenotypes observed in patients carrying *FLNA* defects, indicating that impaired filamin-A is likely responsible for several human genetic diseases. The underlying pathological mechanisms of these diseases are complex and might be attributed to the loss of FLNA partner bindings or through aberrant FLNA protein interactions. Nevertheless, the effect of most of patient mutations on FLNA partner interactions remains to be elucidated at the molecular level.

### Filamin-A in cytoskeleton reorganization and cell shape determination

The cytoskeleton provides rigid structural support that is responsible for maintaining cell shape. Somewhat paradoxically, this rigid network is also highly active and dynamic, which endows the cells with plasticity and adaptability to respond to stimuli from the surrounding environment. As an actin binding protein, filamin-A plays an essential role in these processes. Filamin-A is responsible for cross-linking actin filaments into orthogonal networks. These Filamin-A-actin networks possess active and reversible organizational properties, which protect cell from various shear stresses [44-46]. As shown in Figure 1, the unique structure of filamin-A, homodimer and multiple actin binding sites (ABD and Rod 1 domain), forges its high avidity binding to F-actin [47]. FLNA hinges alternatively confer flexibility to the filamin-A molecule [44]. The conformation of Rod 2 region also modulates the interaction of FLNA with multiple binding partners (Table 1), which allow the binding affinity of FLNA to actin filaments to be finely tuned to the arrangement of F-actin which varies throughout the cytoplasm [48-51]. Furthermore, filamin-A has been shown to regulate intermediate filament systems via the interaction with vimentin, a component of intermediate filaments [52,53]. These conserved structural functions allow filamin-A to be a highly dynamic mediator of cytoskeleton reorganization.

**Table 1:** A partial list of Filamin-A interacting partners

**Interacting partners**	Binding sites^*^	Approach^**^	**Significance**	**Reference**
**Cytoskeleton and cell shape maintenance**				
F-actin	ABD, Rod-1	b	3D F-actin networks with unique mechanical and physiological properties	[47,54]
Calmodulin	ABD	b	Regulates F-actin binding *in vitro*	[55]
R-Ras	3	b, c	Enhances integrin activation and maintains endothelial barrier	[56,57]
Syk	5	b, c	Supports ITAM-mediated receptor signaling in platelet	[58]
Vimentin	1-8	c	Vimentin phosphorylation, cell surface expression of β1 integrins and cell spreading on collagen	[52,53]
Supervillin	8–10, 20–22	a	Cell spreading	[59]
**Membrane and membrane associated proteins**				
Dopamine D2 and D3 receptors	19	a, b, c	Stabilizes β-arrestins-filamin-A complex	[60,61]
Pro-Prion	10,16–18, 20, 21, 23	b, c	Enhances the binding of filamin-A with β1 integrin, and promotes cell spreading and migration in melanoma	[62,63]
GPI bα (CD 42b)	17	b, c	Intracellular trafficking and maintains the size of platelets	[64,65]
β Integrins	21	c	Adhesion, mechanoprotection and competing binding site with talin to regulate integrin activation	[66,67]
Tissue factor	22-24	a, b	Supports cell spreading and migration	[68]
CEACAM 1	23–24	a, b	Reduces cell migration	[69]
Migfilin (FBLP-1)	21	a, b, c	Disconnects filamin-A from integrin and promotes talin-integrin binding	[48,70-72]
Caveolin-1	22-24	a, b	Intracellular trafficking	[73,74]
**Intracellular signaling**				
β-arrestins	22	a, b, c	ERK activation and actin cytoskeleton reorganization	[75]
Wee1	22-24	b, c	Regulates Wee1 expression and promotes G2/M phase progression	[42]
K-RAS	nd	nd	Filamin-A deficiency reduces K-RAS oncogenic potentials	[76]
NIK	nd	b	Mediates the activation of the IKKα/NF-κB cascade through CD28 signaling	[77]
sst2	19-20, 21-24	b, c	Negative control on PI3K pathway	[78]
Androgen receptor	16-19	a, b, c	Required for androgen-induced cell migration	[79,80]
SEK1	22-23	a, b, c	Tumor necrosis factor-alpha signaling	[81]
TRAF2	15-19	a, b, c	Inflammatory signal transduction	[82]
**Small GTP-binding proteins and their regulators**				
Rho/Cdc42/RalA	24	c	Remodeling of cytoskeleton	[83]
ROCK	24	b, c	Remodeling of cytoskeleton	[84]
FilGAP	23	a, b, c	Cell spreading and GAP activation	[17,85]
Trio	23–24	c	GEF for RhoG/Rac1 and RhoA and required for ruffling	[86]
**Nuclear function associated proteins**				
BRCA1	23-24	a, b, c	Facilitates the recruitment of BRAC1 and RAD51 to DNA damage sites and stabilizes the DNA-PK holoenzyme	[87]
BRCA2	21-24	a, b, c	Required for efficient homologous recombination DNA repair and recovery of G2/M phase arrest	[88-90]
RefilinB	15-24	a, b	Stabilizes perinuclear actin actin networks and regulates nuclear shape	[91]
TIF-IA, RPA40	ABD	b	Suppresses ribosomal RNA gene transcription	[92]
TAF1B/mKIAA1093	1-7	a, c	Possible role in rRNA production, protein translation and the organization of centromeres	[93]

Note:

*: This column indicates the domains involved in the respective interactions. Numbers in this column represent the repeat numbers of filamin-A.

**: The approaches used to demonstrate the interaction including, (a) yeast two-hybrid; (b) *in vitro* pull-down; (c) co-immunoprecipitation; or (nd), not determined.

Filamin-A also connects actin networks to plasma membrane. The distinctive shape of a cell depends on the organization of the actin network together with the proteins that connect actin filaments to the plasma membrane. When attached to a planner network of actin filaments, the membrane maintains as a flat surface. However, when the underlying actin network is organized into parallel bundles of actin filaments, the membrane acquires the finger-like shape of cell protrusion, such as lamellipodia, pseudopodia and filopodia. In addition to cross-linking actin filaments into 3D networks, filamin-A interacts with a wide variety of proteins, including transmembrane proteins and signaling molecules (Table 1). Additionally, filamin-A acts an adaptor that mediates the complex connections between the integral membrane proteins and actin filaments, which enables filamin-A to modulate cell shape at specific areas. Moreover, actin filaments are enriched in the cortex of many cells, a narrow zone just beneath the plasma membrane, where the protrusive structures are formed. At both the leading edge and the rear of polarized motile cells, filamin-A can bind actin to influence the nature of cytoplasmic protrusions and retractions [94,95]. Interestingly, it has been shown recently that filamin-A organizes perinuclear actin networks and regulates the nuclear shape via RefilinB (FAM101B) [91].

In addition, Filamin-A acts as a scaffold for signaling molecules that mediate intracellular protein trafficking. Membrane receptors sense and transmit extracellular biochemical signals across cell membranes, which is dependent on the association with the F-actin cytoskeleton. Filamin-A regulates matrix–cytoskeleton signaling by virtue of binding to both actin networks and the cytoplasmic tails of membrane receptors (Table 1). One of the most well-studied interactions is with the integrin family of proteins which have recently been described at the atomic level [48,49]. In response to intergrin binding, the filamin-A molecule is mechanically stretched, which alters partner binding affinity while opening new partner binding sites. It is hypothesized that this phenomenon underlies FLNA’s mechanism for regulating the cytoskeleton. Following integrin binding to extracellular matrix ligands, filamin-A coordinates the actin-remodeling activities of GTPase signaling factors as shown in Table 1, and anchors them in proximity to the cell membrane, leading the formation of lamellipodia and filopodia [10]. Upon stimulation, membrane receptors are continually endocytosed and recycled back to the cell membrane by trafficking [96,97]. While the biochemical pathways of protein trafficking are not completely defined, increasing evidence demonstrates that filamin-A is pivotal for the intracellular trafficking and for deployment of cell β1 integrins on the cell membrane, which are essential for maintaining directed cell migration[53,98,99].

### Filmin-A and cell-matrix interaction

The extracellular matrix (ECM) not only provides essential physical support to cells but also initiates crucial biochemical and biomechanical cues that are required for tissue morphogenesis, tumor invasion, and wound healing [100,101]. However, the mechanisms by which cells recognize and respond to changes in ECM properties are not clear. It is believed that adhesion receptors such as integrins, discoidin domain receptors and syndecans mediate cell to cell communication [102-104]. Upon stimulation by extracellular matrix ligands, intergrins are activated and recruit filamin-A to actin filaments, which facilitate cell spread through matrix-to-cell (outside-in) signals. As a result, filamin-A may enhance integrin–ligand interactions by binding to the cytoplasmic domain of the β1-intergrin subunit through inside-out signaling [98].

Although interactions with adhesion receptors facilitate the interplay between the cell and the ECM, the mechanisms by which filamin-A regulates cell migration are not simply unidirectional. For example, the strong association between integrin and filamin-A impairs migration [105], and the competition binding by talin to replace filamin-A can resume signaling and movement, which altogether underscores the importance of a balance of multiple interactions during migration [66,67,105]. Xu et al. reported that filamin-A regulates focal adhesion disassembly, and that down-regulation of filamin-A enhances the cleavage of focal adhesion proteins [106]. Furthermore, filamin-A has been shown to play a role in ECM degradation and it has been demonstrated that knockdown of filamin-A increases the expression of matrix metalloproteinase-9 (MMP-9) [107] which induces MMP2 activation [108].

### Filamina A and cancer metastasis

It is known than filamin-A interacts with many proteins related to cancer metastasis [17,41,52,83,109,110] (Table 1). Although a large volume of studies associate filamin-A with cancer metastasis, the specific roles of filamin-A during metastatic invasion remain elusive. This enigma is a reflection of the complex nature of metastasis, a multifactorial process which involves tumor cell detachment from the primary sites, followed by tumor cell invasion of the EMC and migration, and finally intravasation, survival in the vasculature, extravasation, and colonization at the secondary sites. Metastasis requires the cancer cells to be able to adapt to different cells shapes, to resist to mechanical stress, and to be highly motile. In addition, it is highly important that the cell is able to re-attach to ECM at secon dary sites. It should come as no surprise that all of these processes are related to the filamin-A cytoskeleton network. Thus, it is conceivable that lack of filamin-A would render the tumor cells less mobile and less invasive, more sensitive to mechanical stress, and result in inefficiency in attachment to ECM at secondary sites to form metastasis colonies. It is also possible that cancer cells with appropriate filamin-A levels would likely have an advantage during metastasis. Alternatively, during the early stages of tumorigenesis, *in situ* cancer cells need to be detached from the original sites to initiate metastasis. It stands to reason that filamin-A may be required for the cancer cells to remain attached to the original site, and that mis-regulation or loss of filamin-A may increase the risk of initiation of cancer metastasis. Due to the dual roles of filamin-A in the regulation of cancer cell mobility and cell interaction with the extracellular matrix, it is unsurprising that inconsistent results were reported on filamin-A’s role in cancer metastasis (see a summary in Table 2).

**Table 2:** Summary of literature of filamin-A in cancer metastasis

**Research system**	**Observations**	**Reference**
**Literatures reported the role of filamin-A in facilitating metastasis and cell locomotion**		
Meckel-Gruber syndrome patient	Filamin-A interacts with the cytoplasmic domain of meckelin, a transmembrane receptor, which is essential for neuronal migration and Wnt signalling	[111]
Hepatocellular carcinoma (HCC)	Comparative proteomics revealed that high level of filamin-A expression is associated with increased metastatic potentials of HCC cells.	[112]
Cancer tissues	By using a newly developed antibody that recognizes secreted variant of filamin-A, gradually increased levels of filamin-A was detected in normal breast tissue, localized and invasive breast cancer, which is associated with cancer progression.	[113]
Prostate cancer cell and tissue microarray	Filamin-A proteolysis results in nuclear localization of 90 kDa fragment, which is associated with decreased cancer metastasis, while elevated cytoplasmic levels of filamin-A was associated with enhanced metastatic potential	[114]
FlnA-knockdown rats	Filamin-A deficiency results in the abnormal migration, and then further causes disorganization of radial glia, which is the leading cause of PH pathogenesis.	[115]
NIH3T3 and HT1080 cells	Interaction of filamin-A with androgen receptor is essential for integrin β1 and FAK activation and cell migration induced by androgen stimulation	[79]
M2 and A7 melanoma cells	Filamin-A functions to stabilize cortical actin in vivo and is required for efficient cell locomotion	[16]
FlnA null mouse platelets	The interaction between FlnA and Syk regulates ITAM- and ITAM-like-containing receptor signaling which is essential for platelet spreading	[58]
M2 melanoma cells	R-Ras regulates migration through an interaction with filamin A in melanoma cells	[57]
EK-293 cells	Filamin A interacts with vimentin to regulation of cell adhesion to collagen through recycling beta1 integrins to cell membrane	[52,53]
Melanoma and breast cancer cells and breast cancer TMA	Filamin-A deficiency in melanoma and breast cancer cells reduces not only cell motility and invasiveness, but also spontaneous and systemic metastasis in nude mouse xenograft. Decreased filamin-A expression levels in cancer cells are associated with better survival of distant metastasis-free in breast cancer patients.	[116]
**Literatures reported the role of filamin-A inhibiting metastasis**		
Human fibrosarcoma cells	Filamin-A deficiency increases matrix metalloproteinase (MMP) activity and induces MMP2 activation, enhancing the ability of cells to remodel the ECM and increasing their invasive potential	[108]
HT1080 and Jurkat cells	Filamins play a role in cell migration and spreading through the interactions between filamins and transmembrane or signaling proteins, which is mediated at least in part by repeat 19 to 21.	[117]
A7 melanoma cells	Migfilin acts as a molecular switch to disconnect filamin from integrin for regulating integrin activation and dynamics of extracellular matrix-actin linkage.	[71]
Hematopoietic cell	ASB2 may regulate hematopoietic cell differentiation by modulating cell spreading and actin remodeling through targeting of filamins for degradation	[48,118,119]
Chinese hamster ovary cells	Tight filamin binding restricts integrin-dependent cell migration by inhibiting transient membrane protrusion and cell polarization.	[105]
A7 and M2 cells	Co-expression of CEACAM1-L and filamin A lead to a reduced RalA activation, focal adhesion turnover and cell migration	[69]
Primary melanoma cell line	Wnt5A activates calpain-1, leading to the cleavage of filamin A, which results in a remodeling of the cytoskeleton and an increase in melanoma cell motility.	[120]
ErbB2 overexpressed breast cancer cells and Breast TMA	Filamin-A deficiency in ErbB2-breast cancer cells reduces FAK turnover and cell motility. Down-regulation of filamin-A in stromal and base membrane is associated with breast cancer progression and invasive lymph node status	[106]

In support of the hypothesis that filamin-A promotes cancer metastasis, a comparative proteomic study has identified that high levels of filamin-A were correlated with increased metastatic potential of hepatocellular carcinoma [112]. Using a three-dimensional migration approach, Quite et al. found that filamin-A is required for podosome stabilization, podosome rosette formation, extracellular matrix degradation, and three-dimensional mesenchymal migration [121]. Li et al. identified the interaction of pro-PrP with filamin-A and the binding enhances association between FLNA and integrin β1, which promotes cell spreading and migration and further contributes to melanomagenesis [62]. Recently, we have shown that knockdown of filamin-A in melanoma C8161 cells reduced metastasis in xenograft mouse models, and that filamin-A inhibition reduced the mobility and invasiveness of breast cancer cell lines that do not over-express ErbB2 *in vitro*[116]. These studies support the model that filamin-A expression is likely responsible for remodeling cancer cell shape and mobility which is integral for initiating metastasis.

Filamin-A readily undergoes proteolysis at its two hinge regions to generate 170 kDa, 150 kDa, 120 kDa, 110 kDa and 90 kDa of major fragments. Based on IHC on human prostate tissue microarray, Bedolla et al. found that filamin-A proteolysis is associated with a reduction of metastatic potential of prostate cancer. The prostate cancer metastasis correlates with cytoplasmic localization of full-length filamin-A but not nuclear filamin-A fragments [114]. It was further suggested that metastasis may be prevented by cleavage and subsequent nuclear translocation of this protein [114]. Castoria et al. recently reported that the interaction of filamin-A with androgen receptor (AR) at the cytoskeleton can be rapidly induced upon stimulation by androgen. Filamin-A/AR complex further recruits integrin beta 1, activating a cascade that drives cell migration [79]. Cells expressing mutant AR that lack a filamin-A interacting domain fail migrate in response to androgen stimulation [79].

At the same time, a few reports suggest that reduction of the full-length filamin-A promotes metastasis by a different mechanism. Baldassarre et al. showed that knockdown of filamin-A in fibrosarcoma increased matrix metalloprotease 2 activity and activation, enhanced the ability of cells to remodel the ECM, and increased cellular invasive potential without significantly altering two-dimensional random cell migration [108]. In addition, using breast cancer cells that over-express ErbB2, Xu et al. reported that knockdown of filamin-A promoted cleavage of focal adhesion in cancer cells and stimulated cancer cell migration, invasion, and metastasis. The same authors also reported that local breast cancers during early development have a higher levels of filamin-A than late counterparts [106]. Although the question of whether the filamin-A levels witnessed in these cancer tissues were inclusive of surrounding ECM among the *in situ* carcinoma remains elusive, this study suggests that loss of filamin-A enhances FAK turnover, which results in the disrupting cancer cell attachment to the EMC. Furthermore, O’Connell et al. demonstrated that Wnt5a-activated calpain 1 is able of cleaving filamin-A, which causes cytoskeleton remodeling and enhances melanoma cell motility [120].

The apparent discrepancies among the literature strongly indicate that levels of filamin-A in the cancer cells may not be the sole indicator of predicting whether a cancer is more or less metastatic. The type of cancer cells (such as carcinoma verses fibrosarcoma), concurrent expression of other relevant genes in the cancer cells (such as ErbB2), their interactions with the extracellular environment, and the proteolysis of filamin-A also appear to be major collaborating factors for filamin-A mediated metastatic invasion.

### Filamin-A in cell signaling and cancer progression

In addition to the regulation of metastasis, filamin-A is also involved in other aspects of tumor progression. The interaction between filamin-A and R-RAS has been suggested to be responsible for maintaining the endothelial barrier function [56]. Knockout of mouse *FlnA* significantly reduces the oncogenic properties of K-RAS, including lung tumor formation, proliferation of K-RAS expressing fibroblasts, and the activation of the downstream signaling molecules such as ERK and AKT [76]. Found in most cases of infant acute lymphoblastic leukaemia (ALL) and acute myeloblastic leukaemia (AML), the fusion of MLL and its partner gene leads to a gain of function of the MLL gene, which affects the differentiation of the hematopoietic pluripotent stem cells or lymphoid and myeloid committed stem cells [122]. *FLNA* was also identified as a new partner gene fused to MLL [123]. Recently, Muscolini et al. identified the interaction between filamin-A and NF-κB inducing kinase (NIK), and found that filamin-A is essential for mediating the activation of the IKKα/NF-κB cascade through CD28 signaling [77].

Interaction of FLNA with membrane transforming receptors has also been reported. Najib et al. found that filamin-A can compete with PI3K-p85 binding to the G protein-coupled receptor (GPCR) sst2 to negatively regulate PI3K signaling [78]. It has also been demonstrated that filamin-A modulates signaling events and cellular responses induced by external stimuli, including EGF receptor [124] and tumor necrosis factor (TNF) receptor-associated factor 2 (TRAF2) [82].

Although *FLNA* mutations can be the cause of human genetic disease, increased cancer incidence has not been observed in these patients. Furthermore, filamin-A is dispensable for cell-autonomous survival and loss of filamin-A expression or depletion of filamin-A with RNAi does not cause cell death or not impede growth [88,125-127]. Nevertheless, disruption of filamin-A function may contribute to the biology of cancers and provide the tumor cells with a growth advantage. Therefore, further studies are required to address whether anomaly in *FLNA* can initiate tumorigenesis.

### DNA damage response and nuclear functions of Filamin-A

In the past two decades, a striking discovery related to filamin-A is its nuclear functions, especially in DNA damage response. In 2001, Yuan and Shen first reported the interaction between filamin-A and BRCA2, a critical protein involved in DNA damage repair [89]. Coincidentally, Velkova et al. reported interaction of filamin-A with BRCA1, another DNA repair protein, in 2010 [87]. It has been demonstrated that inhibition of filamin-A moderately inhibited homologous recombinational DNA repair, sensitized cells to ionizing radiation and cisplatin, and prolonged the recovery of G2 arrest following irradiation [90]. Velkova first suggested a role of filamin-A in non-homologous end joining repair of the DNA double strand breaks [87] and has since fundamentally established a role of filamin-A in DNA damage response through the interactions with BRCA1 and BRCA2 [87,88,90,126,127]. Because DNA damage response signaling and DNA repair play a critical role in the maintenance of genomic integrity [128], these studies also raised a possibility that mis-regulation of filamin-A function may contribute to the initiation of tumorigenesis.

It is likely that filamin-A acts in DNA damage as auxiliary factor to provide nuclear matrix support for the repair machinery instead of serving as an enzyme directly involved in processing DNA damage. Perhaps due to this indirect role in DNA damage response, the specific consequence of filamin-A inhibition in cell sensitivities to DNA damage depends on the type of damage or how the DNA damage is induced. For example, although inhibition of filamin-A sensitizes cells to radiation, cisplatin, and bleomycin, it surprisingly also confers a resistance to topo-isomerse poisons [126].

It has also been demonstrated that filamin-A interacts with RefilinB (FAM101B) to organize perinuclear actin networks and regulate nuclear shape, which is essential for normal nuclear function maintenance [91]. Deng et al. also demonstrated that filamin-A is a nucleolar protein that suppresses ribosomal RNA gene transcription [92]. In addition, Oiu et al. identified two nuclear binding partners of filamin-A, TAF1B and mKIAA1093, from mouse embryonic cDNA libraries, which further endorses the proposed nuclear function of filamin-A [93].

### Filamin-A as a biomarker and potential target for cancer therapy

Due to its functions in the control of cell mobility, cell-ECM interactions, cell signaling, and DNA damage response, it is conceivable that filamin-A may be developed as a biomarker for cancer diagnosis and outcome prediction. When compared with normal tissues, increased expression of filamin-A was observed in various types of cancers. In some cases, different stages of cancers have varied level of filamin-A expression. Thus, when compared with normal tissues, filamin-A expression may be considered as an alternative diagnostic marker. In addition, variable expression levels of filamin-A protein among different cancers may serve as a useful tool for individualized therapy and outcome prediction.

The correlation of filamin-A expression patterns in different stages of cancer tissues and cancer outcomes has been reported. Uhlen et al. employed antibody-based proteomics to evaluate the expression and localization of FLNA profiles in 48 normal human tissues and 20 different cancers [129]. They found that ~50 % of the colorectal cancer had moderate to high levels of filamin-A, whereas filamin-A was undetectable in normal colon glandular cells. Similarly, the expression of filamin-A was increased in pancreatic cancer, while in normal pancreas, exocrine ductal cells had low to moderate levels of filamin-A [129]. A Gene profiling approach was also employed to assess filamin-A expression in human cancer. Up-regulation of filamin-A was detected in salivary gland adenoid cystic carcinoma [130], peripheral cholangiocarcinomas [131], human glioblastomas [132] and in pancreatic cancer [133]. On the contrary, under-expression of filamin-A was also observed in some types of cancers, including human bladder cancer [134,135] and colon adenocarcinoma [136]. These reports suggest that, tumor tissues may have different expression levels of filamin-A from the surrounding normal tissue, and thus FLNA may serve as a useful diagnostic marker.

As discussed in early sections, various reports have implicated that the levels of filamin-A expression in the cancer cells may be correlated with the cancer metastatic potential and sensitivities to therapeutic DNA damage agents. It is conceivable that the level of filamin-A in cancer tissue can be developed as an alternative marker for outcome prediction and individualized therapy. As we previously discussed, the role of filamin-A in DNA damage sensitivity is dependent on the type of DNA damage [126]. A further implication of these findings is that expression level of filamin-A in cancer cells may be exploited as a biomarker to predict the effectiveness of different types of therapeutic DNA damage agents to the cancer subtype [126].

The role of filamin-A in DNA damage response presents an intriguing possibility that targeting filamin-A may be useful for cancer therapy. In a panel of human melanoma cell lines established from cancer patients, a correlation between filamin-A expression and drug sensitivity was observed, i.e. the lower filamin-A level the cell line had, the more sensitive the cell was to bleomycin and cisplatin treatment [127]. In addition to chemotherapeutic drugs, filamin-A deficiency sensitizes melanoma and breast cancer cells to ionizing radiation [88,89]. These data was further confirmed by xenograft animal models, which demonstrated that tumors generated from filamin-A deficient cells displayed a better response to chemotherapy [127]. In addition, Nallapalli et al. found that filamin-A played an important role during lung tumor growth [76]. These studies predict that inhibition of filamin-A sensitizes cancer cells to specific therapeutic DNA damage agents. Considering the metastatic role of filamin-A in cancer cell mobility and re-attachment to secondary sites, it is also conceivable to predict that inhibition of filamin-A would also reduce distant cancer metastasis. Thus, filamin-A may also be an ideal therapeutic target for metastasis control.

Small molecules to block filamin-A function have been attempted. PTI-125 is novel compound binding to filamin-A with 200 femtomolar affinity, which disrupts the toxic signaling of amyloid-β42 (Aβ42), reducing amyloid-related Alzheimer’s disease pathogenesis [137]. The same research group also designed filamin-A peptide fragments containing the Ultra-low-dose naloxone (NLX) binding site, which blocked the protective effect of NLX on both the MOR–Gs coupling and downstream cAMP accumulation induced by chronic morphine, presumably by interfering with NLX’s binding to filamin-A [138]. Considering that the dimerization of filamin-A at the C-terminus is a common feature for filamin-A function, we propose that disruption of the filamin-A dimer formation could be a valuable approach.

In addition to simply inhibiting filamin-A expression, alternative strategies to disrupt the filamin-A functions have been suggested. Because filamin-A cleavage has been shown to be associated with reduced metastasis, Bedolla et al. suggest that strategies to induce filamin-A cleavage may be developed to reduce cancer metastasis [114]. This strategy may be feasible because it is known that filamin-A proteolysis by calpains is regulated by AKT-dependent phosphorylation at serine 2152 [139], and treatment with PI3K inhibitor that abrogates AKT activity induces filamin-A cleavage and localization of the cleavage product into nucleus [140].

**Figure 2:**
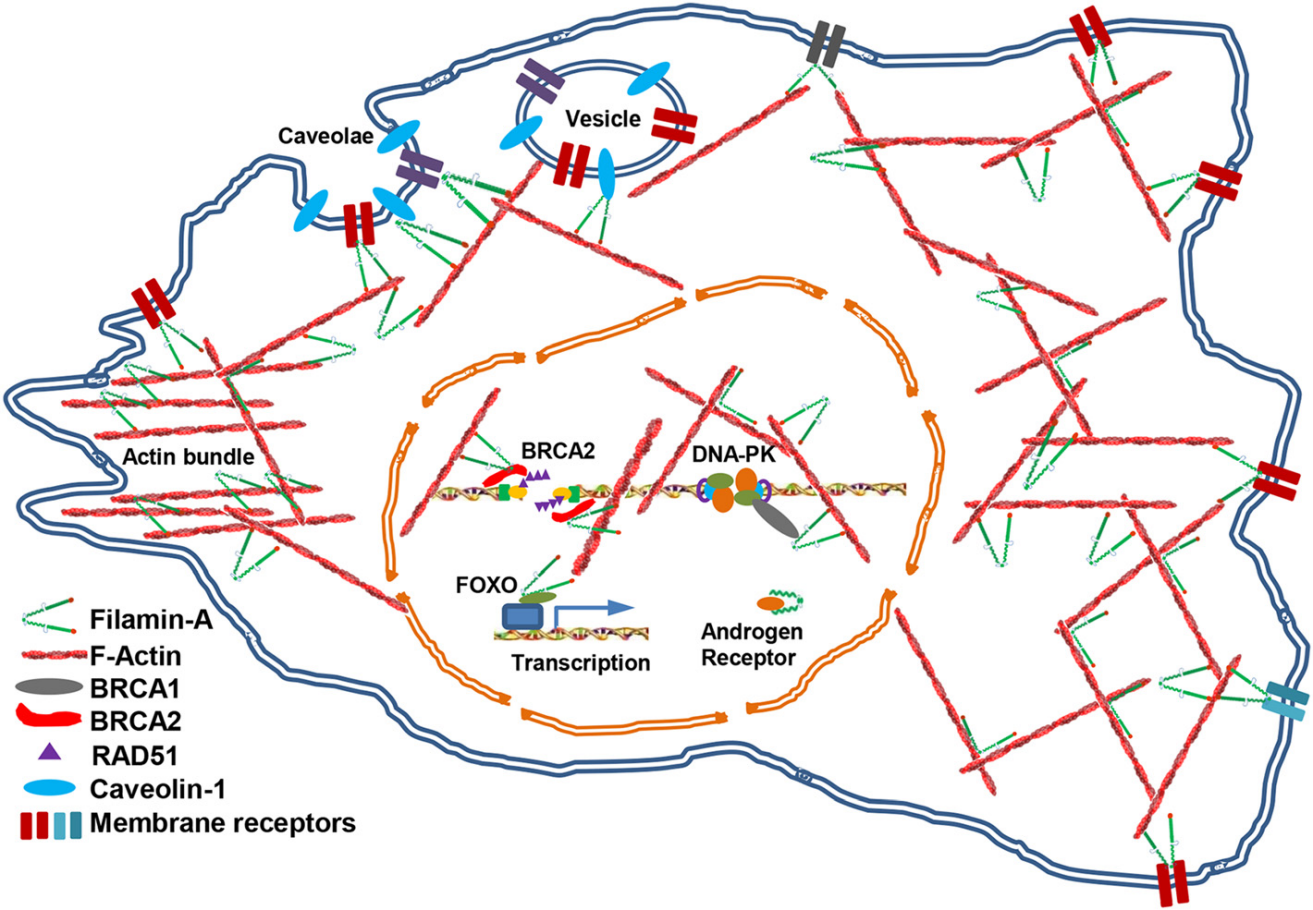
**Schematic presentation of filamin-A functions.** Through the interactions with its binding partners, filamin-A is endowed with versatile cellular functions, including maintenance of dynamic F-actin networks and regulating cell shape; mediating the communication between cytoskeleton and ECM; acting as a scaffold for cell signaling to regulate cell motility; facilitating intracellular trafficking and promoting membrane protein recycling; regulating RNA transcription through interactions with transcriptional factors and RNA polymerase machinery; modulating nuclear receptor signaling through the binding with androgen receptor; and mediating DNA damage response through interactions with BRCA1, BRCA2

In summary, due to its multiple functions as a scaffolding protein, filamin-A integrates intracellular signaling and mediates a variety of cellular processes (Figure 2). Its functions in cancer, especially in metastasis and sensitivity to DNA damage, may be exploited for cancer therapy and outcome prediction. However, the specific consequence of filamin-A inhibition in metastasis and DNA damage sensitivity is likely dependent on the cancer stages and types of DNA damage agents. Extensive studies are required to fully develop filamin-A as a valid cancer marker and therapeutic target.

## Competing interests

The authors declare that they have no competing interests.

## Authors’ contributions

JY drafted the first version of the manuscript and figures; SH amended the first draft; ZS finalized the manuscript and figures. All authors read and approved the final manuscript.
